# Plasma circulating microRNAs associated with blood-based immune markers: a population-based study

**DOI:** 10.1093/cei/uxad126

**Published:** 2023-11-09

**Authors:** Samantha Leonard, Irma Karabegović, M Arfan Ikram, Shahzad Ahmad, Mohsen Ghanbari

**Affiliations:** Department of Epidemiology, Erasmus MC University Medical Center, Rotterdam, The Netherlands; Department of Epidemiology, Erasmus MC University Medical Center, Rotterdam, The Netherlands; Department of Epidemiology, Erasmus MC University Medical Center, Rotterdam, The Netherlands; Department of Epidemiology, Erasmus MC University Medical Center, Rotterdam, The Netherlands; Department of Epidemiology, Erasmus MC University Medical Center, Rotterdam, The Netherlands

**Keywords:** immune markers, neutrophil-to-lymphocyte ratio, platelet-to-lymphocyte ratio, microRNAs, gene regulation

## Abstract

MicroRNAs (miRNAs) are small non-coding RNAs that post-transcriptionally regulate gene expression and different immune-related pathways. There is a great interest in identifying miRNAs involved in immune cell development and function to elucidate the biological mechanisms underlying the immune system, its regulation, and disease. In this study, we aimed to investigate the association of circulating miRNAs with blood cell compositions and blood-based immune markers. Circulating levels of 2083 miRNAs were measured by RNA-sequencing in plasma samples of 1999 participants from the population-based Rotterdam Study collected between 2002 and 2005. Full blood count measurements were performed for absolute granulocyte, platelet, lymphocyte, monocyte, white, and red blood cell counts. Multivariate analyses were performed to test the association of miRNAs with blood cell compositions and immune markers. We evaluated the overlap between predicted target genes of candidate miRNAs associated with immune markers and genes determining the blood immune response markers. First, principal component regression analysis showed that plasma levels of circulating miRNAs were significantly associated with red blood cell, granulocyte, and lymphocyte counts. Second, the cross-sectional analysis identified 210 miRNAs significantly associated (*P* < 2.82 × 10^−5^) with neutrophil-to-lymphocyte ratio (NLR), platelet-to-lymphocyte ratio (PLR), and systemic immune-inflammation index. Further genetic look-ups showed that target genes of seven identified miRNAs (miR-1233-3p, miR-149-3p, miR-150-5p, miR-342-3p, miR-34b-3p, miR-4644, and miR-7106-5p) were also previously linked to NLR and PLR markers. Collectively, our study suggests several circulating miRNAs that regulate the innate and adaptive immune systems, providing insight into the pathogenesis of miRNAs in immune-related diseases and paving the way for future clinical applications.

## Teaser

MicroRNAs (miRNAs) are emerging as post-transcriptional regulators of gene expression in various biological processes, spurring efforts to identify miRNAs implicated in immune cell development and function, to elucidate the biological mechanisms underlying the immune system, its regulation, and disease. This study measured circulating levels of miRNAs by RNA-sequencing in plasma samples of 1999 participants from the population-based Rotterdam Study. Together, we found several circulating miRNAs with the potential to regulate the innate and adaptive immune systems, providing insights into the pathogenesis of miRNAs in immune-related diseases and paving the way for future clinical applications.

## Introduction

Serum levels of blood cells, such as neutrophils, platelets, and lymphocytes, indicate an immune response and provide a source of phenotypic immunity [1]. Due to this, the immune response cells have been presented as possible prognostic markers for chronic diseases represented through ratios. The markers consist of the neutrophil-to-lymphocyte ratio (NLR), the platelet-to-lymphocyte ratio (PLR), and the systemic immune inflammation index (SII) [2], which integrates peripheral lymphocyte, neutrophil, and platelet counts into a single indicator. The NLR, PLR, and SII represent both adaptive and innate immunity by incorporating both types of white blood cells into their ratios and index, which may be reflective of the immune system’s role in complex diseases such as Alzheimer’s [3], atherosclerosis [4], cancer [5], and cardiovascular disease [6, 7].

In recent years, small non-coding RNA molecules called microRNAs (miRNAs) have been identified as potential regulators of immunity, through the development and differentiation of adaptive and innate immune response cells [8, 9]. These small non-coding RNAs pair with messenger RNAs (mRNAs) to direct post-transcriptional repression or mRNA destabilization [10]. miRNAs can repress the expression of specific genes by complementary binding to the target mRNAs, thus preventing translation into a protein or promoting mRNA decay [11, 12]. Epigenetic and genetic defects in the miRNAs biogenesis or structure may disrupt this normal function, which is shown to influence the risk of different diseases through gene regulation [13]. Furthermore, due to the nature of miRNAs, such as their regulation of multiple target genes and stability in easily accessible bodily fluids, such as the bloodstream, they are suggested as potential biomarkers or even therapeutic targets for complex diseases [5, 14–16]. However, the specific pathology of immune regulation via miRNAs has yet to be completely understood.

Earlier studies have illustrated the involvement of miRNAs in the regulation of the immune system and disease status, thus signaling their potential as therapeutic targets and/or biomarkers for immune-related diseases [9, 10, 12, 13, 17–25]. These studies have generally used a subset of miRNAs, conducted in a case-control setting with small sample sizes, and do not investigate the potential role of miRNAs in immune regulation by testing blood-based immunity markers such as NLR, PLR, and SII. These markers are believed to represent immunity more comprehensively in comparison to more traditional markers such as C-reactive protein (CRP) or erythrocyte sedimentation rate [2, 21, 26]. While it has been shown that levels of inflammatory markers are typically elevated in patients with chronic diseases, the mechanisms that influence NLR, PLR, and SII in chronic disease are largely unknown [11]. The emerging role of miRNAs in regulating immune response through blood-based immune markers could provide more insight into the development of immune-related chronic diseases and aid in their identification and treatment [5]. Additionally, while blood cell counts have been shown to influence plasma levels of miRNAs, little is known about the cross-talk between various blood cell types and circulating miRNA levels or miRNA release mechanisms in immune pathology [9].

The main objective of this study was to investigate the association between plasma levels of circulatory miRNAs and innate and adaptive immunity markers (NLR, PLR, and SII) in a population-based setting. Thus, regression models were conducted to identify immune marker-associated miRNAs, followed by miRNA target gene analysis to differentiate the potential direction of association. The secondary research aim was to determine if plasma levels of cell-free miRNAs may be influenced by the specific cell type from which they are secreted. To achieve this objective, we additionally tested the association of blood cell compositions with miRNA levels using principal component regression.

## Materials and methods

### Study population

This study was conducted within the Rotterdam Study (RS), which is a large prospective population-based cohort study in the Ommoord district in the city of Rotterdam, the Netherlands. Starting in 1989, the Rotterdam Study is an ongoing study investigating the occurrence, risk factors, and progression of chronic diseases in the middle-aged and elderly. The first cohort (RS-I), containing 7983 participants, was extended in 2000 with a second cohort (RS-II) of 3011 participants, and again in 2006 with a third cohort (RS-III) of 3932 participants. The most recent cohort (RS-IV) started with almost 4000 participants. This resulted in a study population of more than 18 000 individuals. Enrolled participants, aged 45 and older, were examined at study entry and follow-up visits every 3–5 years. A detailed description of the RS has been previously described [18].

**Figure 1.**
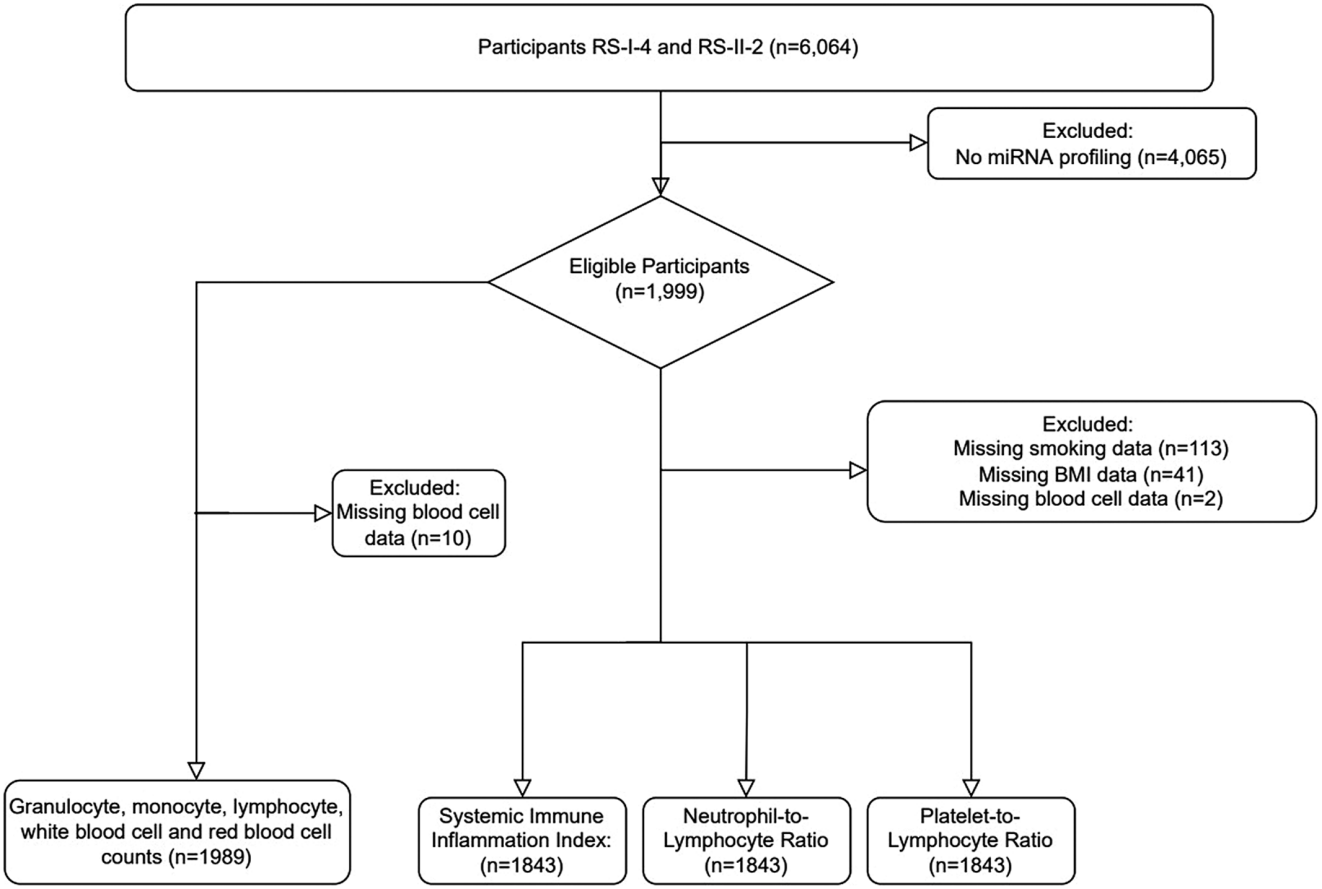
Flowchart depicting the inclusion and exclusion of study participants. In total 1989 participants were included in the cross-sectional study between blood cell compositions and miRNAs. 1843 participants were included in the cross-sectional study between immune markers (SII, NLR, and PLR) and miRNAs

The current study includes 1000 participants from the fourth visit of RS-I and 999 participants from the second visit of RS-II, randomly selected, from which circulatory miRNA levels from plasma were measured (*n* = 1999). From these 1999 participants, 156 were excluded due to missing data for smoking, BMI, and blood cell counts, giving a total of 1843 participants with immune marker data included in the present study. For the analysis of miRNAs and blood cell compositions, 10 participants were excluded due to insufficient baseline data for blood cell compositions. A total of 1989 individuals had data on granulocyte, monocyte, lymphocyte, red blood cell, and white blood cell counts and were included in this study, as depicted in Fig. 1.

### MicroRNA expression profiling

Blood samples were obtained in containers treated with EDTA, followed by centrifugation. Subsequently, plasma was aliquoted and frozen at −80°C in accordance with standard procedures to ensure the preservation and integrity of the samples [18]. Plasma levels of circulatory miRNAs were then measured using the HTG EdgeSeq miRNA Whole Transcriptome Assay (WTA) (HtG Molecular Diagnostics, Tuscon, AZ, USA) and with Illumina MiSeq sequencer (Illumina, San Diego, CA, USA). Methods of plasma miRNA measurement have been previously described in detail [18]. In brief, the WTA characterizes the expression levels of 2083 miRNAs and measures the expression of 13 housekeeping genes as controls, with quantification based on counts per million and standardized by log2 transformation. Plasma samples are then tagged individually using molecular barcodes, pooled, and sequenced [14]. Out of 2083 miRNAs, 591 miRNAs were well expressed in plasma and selected using the lower limit of quantification.

### Collection of blood cells and calculating immune markers

Immediately after blood samples were drawn, full blood count measurements were performed using the COULTER® AcT diff2™ Hematology Analyzer (Beckman Coulter, San Diego, California, USA). Measurements included absolute granulocyte, platelet, lymphocyte, monocyte, red blood cell (RBC), and white blood cell (WBC) counts in 10^9^/L (3). The neutrophil-to-lymphocyte ratio (NLR) was calculated using absolute peripheral granulocyte, as a proxy for neutrophil count (N), and lymphocyte (L) blood counts using the formula NLR = N/L [2]. The platelet-to-lymphocyte ratio (PLR) was calculated using peripheral platelet (P) and lymphocyte (L) blood counts, using the formula PLR = P/L. The systemic immune-inflammation index (SII) was calculated using peripheral platelet (P), granulocyte (N), and lymphocyte (L) blood counts, using the formula SII = P * N/L [2]. These three immune markers are ratios or indexes and thus have no unit. The immune marker data is calculated and currently available within the RS [18].

### Assessment of covariates

The covariates included in the study were age, gender, smoking status, body mass index (BMI), and the Rotterdam Study sub-cohort. Covariates were selected based on biological plausibility and previous literature [14–16, 27]. Information on smoking status was obtained during home interviews [18] and categorized into never, current, and former. Height (m) and weight (kg) were measured, from which BMI = kg/m2 was calculated. The study participants were from two sub-cohorts of the Rotterdam Study (RS-I-4 and RS-II-2). Detailed descriptions of measurement methods for covariates are available within the Rotterdam Study update paper [18].

### Statistical analysis

We performed natural log transformation on immune response markers (NLR, PLR, and SII) and blood cell counts. Multiple linear regression models were used to examine the relationship between plasma levels of 591 well-expressed circulatory miRNAs and immune markers. The Bonferroni-corrected threshold was calculated based on the number of tests (0.05/(591 × 3) = 2.82 × 10^−5^), including 591 miRNAs and three immune markers. The basic model, Model 1, adjusted for age and sex in the analysis. Model 2 was additionally adjusted for BMI, cohort, and smoking status. Sensitivity analysis was performed by adjusting for red blood cell counts in addition to age, sex, BMI, cohort, and smoking status (Model 3).

Principal component regression (PCR) was performed to investigate the relationship between circulatory miRNAs and the blood cell counts for granulocytes, lymphocytes, monocytes, RBC, and WBC. Principal component analysis (PCA) was first performed on plasma levels of 2083 circulatory miRNAs from which the first two principal components (PC1 and PC2) were used as exposure variables. Linear regression models were used to examine the association of PC1 and PC2 with blood cell counts with a false discovery rate (FDR) < 5% considered as the significant threshold.

All data analyses were conducted using IBM SPSS Statistics version 25.0 for Windows (IBM Corp., Armonk, NY, USA) and R software (R-1.4.1106; R Foundation for Statistical Computing, Vienna, Austria).

### In-silico genetic analysis

An in-silico analysis on miRNA target genes was performed to assess the potential involvement of the NLR and PLR-associated miRNAs in pathways underlying immune-related diseases. To this end, experimentally validated target genes of miRNAs were extracted from a commonly used miRNA target prediction database, miRTarBase [28]. To assess whether these target genes of miRNAs have been previously associated with NLR and PLR, a literature review was conducted using PubMed, and a look-up was performed in the data of the previous genome-wide association study (GWAS) [29] using GWAS catalog [30]. For the GWAS look-up, data on the single nucleotide polymorphisms (SNPs) associated with NLR and PLR (*P* < 5.0 × 10^−8^) with annotations to their respective genes was used [29]. From this, overlapping miRNA target genes were extracted. Further assessment was conducted on whether any of these genes are involved in immune pathophysiology through a look-up in the literature (PubMed).

Finally, we utilized the whole blood gene expression and immune markers data of the Rotterdam Study to further demonstrate a relationship between blood levels of identified miRNA-target genes and the immune markers. The Illumina probe ID’s were extracted for each gene followed by RNA expression extraction from 881 subjects of the RS-III. A detailed description of gene expression data in the Rotterdam Study is provided elsewhere [18]. To investigate the relationship between miRNA-target gene expression and immune markers, we conducted a linear regression analysis using the available data from RS-III on the identified target genes. The Beta, standard error, and *P*-value were computed as measures of association after multiple testing corrections. The Bonferroni-corrected threshold of (0.05/(11 × 3) = 1. 52 × 10^-3^) was calculated based on the number of tested probes.

## Results

### Statistical analysis

Participants who had data on miRNA expression, immune markers, and covariates were included for examining the association of miRNA levels with PLR, NLR, and SII. The characteristics of study participants are shown in Table 1. Of the 1843 participants included in the association of miRNAs and immune markers, 854 were males (42.9%) and 1,135 were females (57.1%). Males had a lower mean age, 63.9 (6.46) years, a higher proportion of former smokers, 68.7%, and a higher mean red blood cell count, 4.88 (0.43), when compared to females, who had a mean age of 64.3 (6.93) years, a lower proportion of former smokers, 41.8%, and a lower mean red blood cell count, 4.60 × 10^9/L (0.40). Furthermore, males had lower mean values for SII, PLR, and BMI, which were 6.08 (0.55), 4.70 (0.45), and 27.5 (kg/m^2^, SE = 3.40) respectively, when compared to females, who had mean values for SII, PLR, and BMI of 6.12 (0.49), 4.83 (0.38), and 27.7 (kg/m^2^, SE = 4.60) respectively. Additionally, females had a lower mean value for NLR, 0.54 (0.41), compared to males, who had a mean value of 0.64 (0.44).

**Table 1.** Characteristics of the study population stratified by sex

	Male (*N* = 792)	Female (*N* = 1051)	Overall (*N* = 1843)
**Age, years**			
Mean (SD)	63.9 (6.46)	64.3 (6.93)	64.1 (6.73)
Median [min, max]	62.3 [55.0, 94.0]	62.4 [55.0, 90.2]	62.4 [55.0, 94.0]
**Smoking**			
Never	141 (17.8%)	461 (43.9%)	602 (32.7%)
Current	107 (13.5%)	151 (14.4%)	258 (14.0%)
Former	544 (68.7%)	439 (41.8%)	983 (53.3%)
**Cohort**			
RS I-4	385 (48.6%)	538 (51.2%)	923 (50.1%)
RS II-2	407 (51.4%)	513 (48.8%)	920 (49.9%)
**Body mass index (unit)**			
Mean (SD)	27.5 (3.40)	27.7 (4.60)	27.6 (4.12)
Median [Min, Max]	27.2 [16.2, 38.8]	27.3 [15.3, 50.3]	27.3 [15.3, 50.3]
**Red blood cell count**			
Mean (SD)	4.88 (0.43)	4.60 (0.40)	4.72 (0.44)
Median [min, max]	4.89 [3.01, 6.08]	4.61 [2.41, 9.30]	4.71 [2.41, 9.30]
**NLR**			
Mean (SD)	0.64 (0.44)	0.54 (0.41)	0.58 (0.43)
Median [min, max]	0.63 [-2.30, 2.18]	0.53 [−2.12, 1.90]	0.58 [−2.30, 2.18]
**PLR**			
Mean (SD)	4.70 (0.45)	4.83 (0.38)	4.77 (0.42)
Median [min, max]	4.72 [1.26, 6.08]	4.83 [1.78, 6.09]	4.78 [1.26, 6.09]
**SII**			
Mean (SD)	6.08 (0.55)	6.12 (0.49)	6.10 (0.52)
Median [min, max]	6.07 [2.55, 8.09]	6.11 [3.25, 8.25]	6.10 [2.55, 8.25]

Continuous variables are presented as mean (SD) and median [min, max] and categorical variables are presented as number (%). NLR, PLR, and SII are log-transformed.

NLR indicates neutrophil-to-lymphocyte ratio; PLR indicates platelet-to-lymphocyte ratio; SII indicates systemic immune-inflammation index.

**Figure 2.**
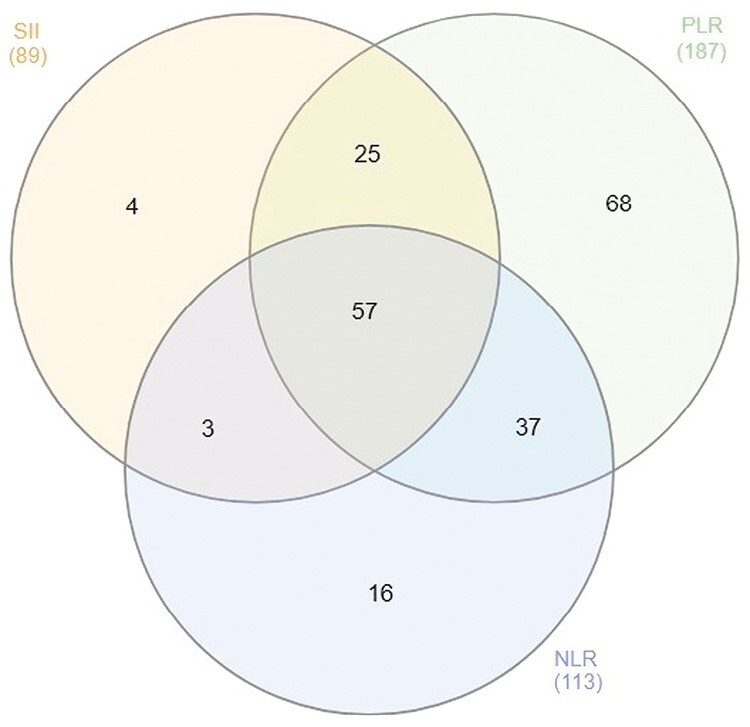
Venn diagram depicting the number of significant miRNAs for each immune marker. There were 89 miRNAs significantly associated with SII, 187 miRNAs significantly associated with PLR, and 113 miRNAs significantly associated with NLR (*P* < 2.82 10^−05^).^§^ ^§^Heberle H, Meirelles GV, da Silva FR, Telles GP, Minghim R. InteractiVenn: a web-based tool for the analysis of sets through Venn diagrams. *BMC Bioinformat* 2015, 16, 169. doi: 10.1186/s12859-015-0611-3

**Figure 3.**
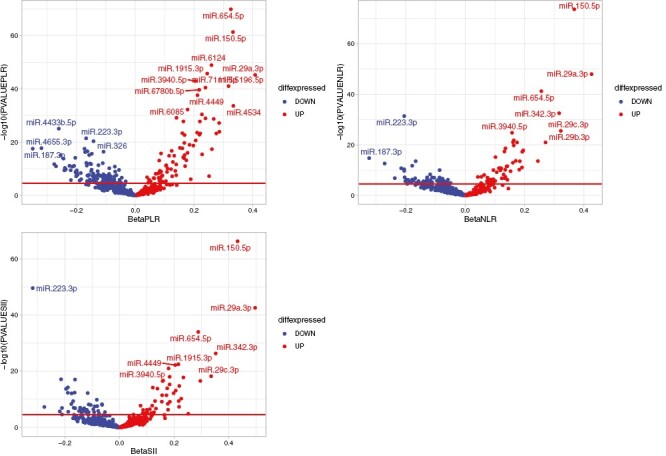
Volcano plots showing correlation between plasma levels of 591 well-expressed miRNAs in plasma and three immune markers PLR, NLR, and SII. The red dots (positive beta) indicate upregulated miRNAs and the blue dots (negative beta) indicate downregulated miRNAs. The red line depicts the Bonferroni-corrected significance threshold based on number of tests at *P* < 2.82 × 10^−05^, with all dots above the significance line representing significant miRNAs

In the multiple linear regression analysis for NLR, PLR, and SII, a total of 210 miRNAs were significantly associated after adjusting for all covariates (full model) and at the Bonferroni-corrected *P* < 2.82 × 10^−5^. Of these, 89 miRNAs were significantly associated with SII, 113 with NLR, and 187 with PLR, as seen in Supplementary Table S1. In addition, we observed association of 57 miRNAs with all three immune markers, as seen in the Venn diagram in Fig. 2. Moreover, volcano plots for PLR, NLR, and SII depict differentially expressed miRNAs in relation to the immune markers (Fig. 3). The number of significant miRNAs did not differ much across the models, with PLR and SII having the same number of significant miRNAs in Model 2. However, NLR had a lower number of significant miRNAs (106) in Model 2 compared to Model 3 (113). The same significant miRNAs remained similar across the three models (Supplementary Table S1).

In the PCA, the two PCs based on 2083 miRNAs accounted for most of the explained variance (9.7% and 4.3%) of miRNA levels, as shown in Supplementary Figs. S1 and S2. The linear regression analysis of PC1 and PC2 with the blood cell counts showed peripheral RBC, lymphocyte, and granulocyte counts to be significantly associated. PC1 and PC2 showed significant association with lymphocyte counts (*β* = −1.41 × 10^−^3, *P* = 3.34 × 10^−^3) and (*β* = 1.99 × 10^−^3, *P* = 6.53 × 10^−^3), respectively. Moreover, PC2 was significantly associated with RBC counts, (*β* = 1.18 × 10^−^3, *P* = 1.79 × 10^−^7), while PC1 was significantly associated with granulocyte counts, (*β* = 1.26 × 10^−^3, *P* = 1.44 × 10^−^2), as illustrated in Table 2. We did not observe a significant association between PCs and white blood cell and monocyte counts (*P* > 0.05).

**Table 2.** Depiction of the regression output for principal components 1 and 2 with the blood cell types

Blood cell type	Exposure variable	Beta	SE	*P*	FDR-corrected *P*
Red blood cell count	PC1	−2.70 × 10^−4^	1.49 × 10^−4^	6.98 × 10^−2^	6.98 × 10 ^−2^
	**PC2**	**1.18 × 10** ^−3^	**2.25 × 10** ^−4^	**1.79 × 10** ^−7^	**8.95 × 10** ^−7^
Lymphocyte count	**PC1**	−**1.41 × 10**^−3^	**4.48 × 10** ^−4^	**3.54 × 10** ^−3^	**8.85 × 10** ^−3^
	**PC2**	**1.99 × 10** ^−3^	**7.29 × 10** ^−4^	**6.53 × 10** ^−3^	**1.09 × 10** ^−2^
Granulocyte count	**PC1**	**1.26 × 10** ^−3^	**5.15 × 10** ^−4^	**1.44 × 10** ^−2^	**1.80 × 10** ^−2^
	PC2	−5.70 × 10^−4^	7.77 × 10^−4^	4.60 × 10^−1^	5.75 × 10^−1^
White blood cell count	PC1	7.63 × 10^−4^	4.70 × 10^−4^	1.04 × 10^−1^	3.42 × 10^−1^
	PC2	8.98 × 10^−4^	7.08 × 10^−4^	2.05 × 10^−1^	3.42 × 10^−1^
Monocyte count	PC1	−1.90 × 10^−4^	6.92 × 10^−4^	7.89 × 10^−1^	7.89 × 10^−1^
	PC2	1.42 × 10^−3^	1.04 × 10^−3^	1.75 × 10^−1^	3.42 × 10^−1^

Red blood cells, lymphocytes, and granulocytes show significant association with one or more principal component. White blood cells and monocytes show no significance.^†,‡^

^†^Simple regression models were used with PC1 and PC2 as the exposure variables. Significant associations are annotated in bold.

^‡^The multiple testing correction, FDR, stands for the false discovery rate.

### Target gene analysis of the identified miRNAs

The predicted target genes of 187 PLR-associated miRNAs, and 113 NLR-associated miRNAs were extracted using the miRTarBase online database [28]. Only experimentally validated (by PAR-CLIP, HITS-CLIP, Luciferase reporter assay, western blot, and/or proteomics) target genes were included to reduce error, which resulted in 7594 genes for 187 NLR-associated miRNAs and 9941 genes for 113 PLR-associated miRNAs (more information in Supplementary Material). In our look-up of previous GWAS data, we found 30 and 83 SNPs to be significantly associated with NLR and PLR and annotated to their respective genes that were among the miRNA target genes [29]. Given no GWAS has been published before for SII, it was excluded from this analysis. In our search in PubMed, we found evidence for miR-1233-3p, miR-149-3p, miR-150-5p, miR-342-3p, miR-34b-3p, miR-4644, and miR-7106-5p to be involved in immune-regulation and immune-related health outcomes [14, 31–37]. The literature search additionally showed previous association of the identified miRNAs with a variety of immune-related diseases [5, 11–15, 17–24, 27, 28, 38]. Among others, these miRNAs were linked to various types of cancers, autoimmune diseases, severe COVID-19, and Alzheimer’s disease.

**Figure 4.**
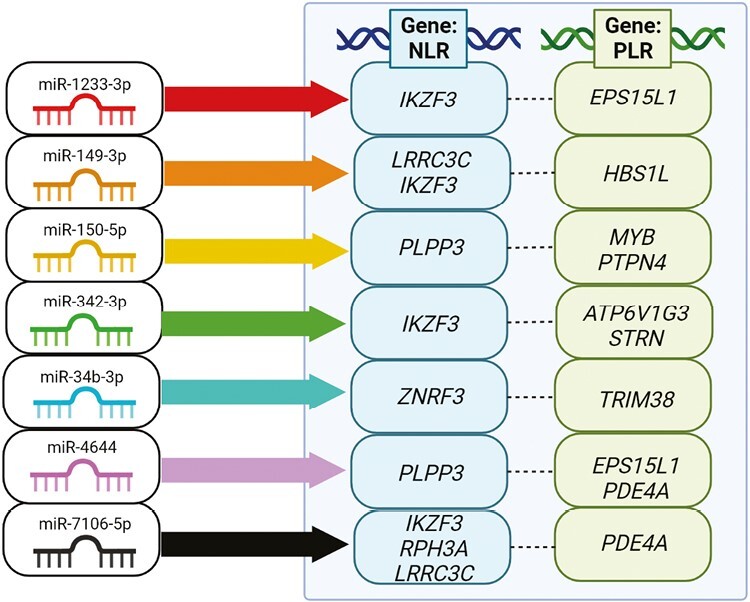
Diagram depicting seven miRNAs that could target both NLR and PLR associated genes. The arrows from the miRNAs depict their target genes, whereas the NLR and PLR gene columns represent previously associated genes Created with BioRender.com

The target gene analysis in particular revealed seven experimentally validated miRNA target genes previously associated with NLR and 26 experimentally validated miRNA target genes previously associated with PLR. Among miRNAs targeting these genes, seven miRNAs were common as regulators of genes associated with both NLR and PLR, as depicted in Fig. 4.

Notably, miR-1233-3p, miR-149-3p, miR-342-3p, and miR-7106-5p could regulate a common target gene *IKZF3*, while the gene *ZNRF3* is a predicted target gene of only miR-34b-3p. Both of these genes have been suggested to be involved in the pathogenesis of immune-related disease [39, 40]. Among the PLR associated genes, *PDE4A* is a target gene of miR-4644 and miR-7106-5p, *EPS15L1* is a putative target of miR-4644 and miR-1233-3p, and both *STRN* and *ATP6V1G3* are target genes of miR-342-3p. Additionally, miR-342-3p could also regulate the expression of *ATP6V1G3*. Lastly, *PTPN4*, a PLR-associated gene, is predicted to be regulated by miR-150-5p. Similarly to NLR-associated miRNA targets, these genes have been thought to regulate immunity and play an essential role in disease pathogenesis [23, 41–49]

Moreover, some of the SNPs from NLR and PLR GWAS are located in intergenic regions near the protein-coding genes *PLPP3, LRRC3C, RPH3A, HBS1L, MYB,* and *TRIM38*. The NLR-associated miRNAs miR-150-5p and miR-4644 target *PLPP3*, miR-149-3p, and miR-7106-5p target *LRRC3C*, while miR-7106-5p targets *RPH3A*. In PLR-associated miRNAs, miR-149-3p could bind to *HBS1L*, miR-150-5p targets *MYB*, and miR-34b-3p targets *TRIM38*. These genes, which have been previously suggested to be regulated by their intergenic regions, have also been shown to regulate pathways that modulate immunity [50–54].

### Statistical analysis of gene expression and immune markers

Lastly, we tested the association between blood expression levels of 10 highlighted miRNA target genes and immune markers in the Rotterdam Study. There were no probe IDs available for *LRRC3C* and *PLPP3,* the genes related to NLR and PLR, in the RNA expression data. Moreover, *ZNRF3* and *TRIM38* genes did not pass the RNA expression quality control (QC). One of three probe IDs for *STRN* did not pass the RNA expression QC, and one of two did not pass for *HBS1L* and *ATP6V1G3*. Yet, our study revealed significant association between several genes, including *IKZF3*, *RPH3A*, *EPS15L1*, *PTPN4,* and *STRN,* and all three immune markers at the stringent Bonferroni corrected threshold of *P* < 1.52 × 10^−3^ (Supplementary Table S2). In addition, *ATP6V1G3* was significantly associated with NLR and SII (*P* < 1.52 × 10^−3^) after correcting for multiple testing.

## Discussion

Within the population-based Rotterdam Study cohort, a cross-sectional analysis was conducted of 591 miRNAs well-expressed in plasma with the blood-based adaptive and innate immunity markers. We found 210 circulating miRNAs that were significantly associated with the immunity markers (NLR, PLR, and SII) after adjustment for potential confounders and multiple testing corrections. Several putative target genes of NLR and PLR-associated miRNAs have been previously linked to the immune markers, suggesting the potential involvement of these miRNAs in immune-related pathways and pathophysiology. These findings may indicate the important role of miRNAs in immune-mediated diseases and their potential as disease biomarkers.

In principal component studies of 2083 circulatory miRNAs and blood cell compositions, we observed the association between RBC and plasma levels of circulating miRNAs. This relationship may indicate that part of the cell-free miRNAs come from lysed RBC (due to hemolysis of erythrocytes) and should be considered in research for disease biomarker discovery using plasma cell-free miRNAs (for example, by adjusting the statistical model for RBC count).

MiRNAs have been previously recognized to be involved in most biological pathways through the regulation of gene expression and are known to be involved in the molecular pathways that modulate inflammation and immunity [17, 20, 21]. For example, miRNAs have been shown to regulate immune checkpoint proteins [32] and mediate chronic inflammation [35]. The 112 NLR-associated miRNAs and the 186 PLR-associated miRNAs have thousands of predicted target genes, which may be dysregulated by alteration in the levels of their respective miRNAs [28]. Pertaining to the predicted target genes of NLR-associated miRNAs, we found seven genes that were associated with NLR in previous GWAS [29]. Likewise, 26 target genes of PLR-associated miRNAs were associated with PLR in GWAS data [29] and thus a likely component of PLR regulation. Notably, we highlighted seven unique miRNAs that target both NLR and PLR-related genes. The gene *IKZF3* is of particular interest as it can be regulated by miR-1233-3p, miR-149-3p, miR-342-3p, and miR-7106-5p, and is thought to be involved in tumorigenesis via T-cell activation and proliferation [39]. Additionally, the target of miR-34b-3p, *ZNRF3*, has been suggested to be involved in tumorigenesis through immune regulation via the Wnt/beta-catenin/TCF signaling pathway [40]. Regarding the PLR-associated genes, *PDE4A* is a predicted target of miR-4644 and miR-7106-5p, which is suggested to regulate levels of cAMP, an immune suppression agent also involved in tumor pathology [41, 42]. The gene *EPS15L1*, an acknowledged target of miR-4644 and miR-1233-3p in PLR, may regulate immune cell development and pathways involved in anti-tumor immunity [43, 44]. Both *STRN* and *ATP6V1G3* are validated targets of miR-342-3p. *STRN* has multiple functional signaling complexes which are reportedly involved in the tumorigenesis of various cancers [45]. miR-342-3p additionally targets *ATP6V1G3*, an integral protein-coding gene for the regulation of membrane transport, which has also been identified as an immunohistochemical marker for different cancers [46]. Lastly, *PTPN4*, a PLR-associated gene, plays a role in immunity via cellular signaling and cell death prevention [23, 47] and has been previously linked to various cancers [48]. MiRNAs have previously been identified as being involved in biological pathways that regulate gene expression, including pathways that modulate immunity and inflammation. The seven unique miRNAs targeting both NLR- and PLR-related genes discovered here further highlight the importance of miRNAs in the regulation of genes involved in immune-related disease.

Furthermore, the annotated miRNA target genes from the analysis of previous GWAS are additionally implicated in inflammatory response [50–54]. For example, *PLPP3* encodes for the integral membrane enzyme lipid phosphate phosphatase 3, which has been suggested to play a regulatory role in immune response, specifically for inflammatory cytokines, leucocyte adhesion, cell survival, and migration in human aortic endothelial cells [50]. *LRRC3C* is believed to affect the risk of inflammatory bowel disease by regulating cell proliferation and apoptosis [51]. *RPH3A* has been proposed to be involved in innate immune response of neutrophil adhesion to endothelial cells [52]. The intergenic region of *HBS1L* and *MYB* genes has been suggested to control erythrocyte, platelet, and monocyte counts, erythrocyte volume, and hemoglobin content [53]. Lastly, the regulatory role of *TRIM38* in innate immunity and inflammation has been extensively studied [54]. These findings provide further insight into the mechanisms of immune pathophysiology via post-transcriptional gene regulation, which could lead to the development of new biomarkers or treatment targets.

Finally, our whole blood gene expression analysis of the highlighted miRNA target genes and immune markers provides additional evidence for the directionality of the observed association. Specified experimentally validated miRNA targets*, IKZF3*, *RPH3A*, *EPS5L1*, *PTPN4,* and *STRN*, were significantly associated with the three immunity markers, and *ATP6V1G3* was associated with NLR and SII, signifying the annotated miRNAs may be involved in regulating immune marker expression. Since there is no existing GWAS for SII, we were not able to investigate target genes for SII-associated miRNAs. The association of the NLR and PLR miRNA gene targets provides further indication that the highlighted genes could play a role in regulating these immune markers, nevertheless, additional research must be conducted.

This study has several strengths which include utilizing a new RNA-sequencing method yielding high coverage of plasma miRNAs, is conducted within a much larger sample size from a population-based cohort, and addresses the involvement of miRNAs in the regulation of innate and adaptive immune markers. The findings from this study suggest the potential of specific miRNAs to be used as biomarkers and/or therapeutic targets in immune-related diseases. However, due to the cross-sectional nature of this study, whether these circulatory miRNAs found in plasma are regulating the immune pathways, or if they are a reflection of the underlying mechanisms causing immune regulation is uncertain. To elaborate on the pathophysiology of the NLR and PLR host genes and potential miRNA target genes, future *in vitro* and/or *in vivo* experimental studies must be conducted. It is beyond the scope of the current study to validate the direction of interaction between the miRNAs and their host and target genes in relation to the immunity markers. With further observational and experimental studies, the exact involvement of identified miRNAs could aid in a more comprehensive understanding of the epigenetic modulation of immunity leading to earlier diagnosis of immune-related diseases and ultimately development of miRNA-targeted therapeutics.

Collectively, this research indicates that specific miRNAs are involved in the regulation of immunity through the post-transcriptional regulation of immune-related genes. Among the identified miRNAs, miR-1233-3p, miR-149-3p, miR-150-5p, miR-342-3p, miR-34b-3p, miR-4644, and miR-7106-5p were associated with both NLR and PLR and may have a prominent regulatory role in immune-related pathways. The downregulation or up-regulation of these miRNAs could reflect an individual’s inflammatory status through NLR and PLR modulation, and therefore, serve as potential biomarkers for immune-related diseases. Future experimental studies are warranted to replicate our findings and assess the causal role of the identified miRNAs in immune regulation, not only to increase our understanding of epigenetic modulation of immunity but also to enhance miRNA biomarker development and RNA-based precision medicine treatments.

## Supplementary Material

uxad126_suppl_Supplementary_Material

## Data Availability

Data can be obtained upon request. Requests should be directed towards the management team of the Rotterdam Study (secretariat.epi@erasmusmc.nl), which has a protocol for approving data requests. Due to restrictions based on privacy regulations and the informed consent of the participants, data cannot be made freely available in a public repository.

## Abbreviations

BMI: body mass index
CRP: C-reactive protein
ESR: erythrocyte sedimentation rate
FDR: false discovery rate
GWAS: genome-wide association study
L: lymphocyte
miRNA: microRNA
mRNA: messenger RNA
N: granulocyte
N: neutrophil
NLR: neutrophil-to-lymphocyte ratio
P: platelet
PC: principal component
PCA: principal component analysis
PCR: principal component regression
PLR: platelet-to-lymphocyte ratio
QC: quality control
RBC: red blood cell
RS: Rotterdam Study
SII: systemic immune-inflammation index
SNPs: single nucleotide polymorphisms
WBC: white blood cell
WTA: Whole Transcriptome Assay
