# Supplementary material for: Plasma circulating microRNAs associated with blood-based immune markers: a population-based study
Source: Clin Exp Immunol. 2023 Nov 9;215(3):251–60. doi: 10.1093/cei/uxad126 (PMC10876108; doi:10.1093/cei/uxad126)
Supplement: uxad126_suppl_Supplementary_Material [file uxad126_suppl_supplementary_material.docx]

Supplementary Material

Table 1.1: Significant miRNAs for SII after Bonferroni corrected threshold of P < 2.82x10^−5^.

| **miRNA** | **Beta** | **se** | **P** |
| --- | --- | --- | --- |
| miR.150.5p | 0.432422866 | 0.023989753 | 5.29E-67 |
| miR.223.3p | -0.317365689 | 0.020628456 | 2.50E-50 |
| miR.29a.3p | 0.496473912 | 0.035049969 | 2.66E-43 |
| miR.654.5p | 0.288511623 | 0.022988752 | 1.01E-34 |
| miR.342.3p | 0.352273223 | 0.032177869 | 4.60E-27 |
| miR.1915.3p | 0.215809605 | 0.021452405 | 3.28E-23 |
| miR.4449 | 0.204226182 | 0.020436147 | 6.21E-23 |
| miR.3940.5p | 0.179605637 | 0.018512216 | 9.75E-22 |
| miR.29c.3p | 0.335523231 | 0.037312292 | 5.88E-19 |
| miR.6780b.5p | 0.183795761 | 0.020563577 | 9.43E-19 |
| miR.3648 | 0.234068684 | 0.02638433 | 1.67E-18 |
| miR.4429 | -0.213590574 | 0.024569275 | 7.66E-18 |
| miR.326 | -0.162411174 | 0.018730414 | 9.25E-18 |
| miR.6085 | 0.160060825 | 0.018673354 | 2.13E-17 |
| miR.29b.3p | 0.296140668 | 0.034695281 | 2.88E-17 |
| miR.6727.5p | 0.157753656 | 0.01850997 | 3.20E-17 |
| miR.155.5p | 0.183683723 | 0.022456934 | 5.27E-16 |
| miR.1237.5p | 0.219043087 | 0.027301168 | 1.82E-15 |
| miR.320d | -0.188586484 | 0.023935487 | 5.61E-15 |
| miR.6798.5p | 0.121923375 | 0.015518165 | 6.66E-15 |
| miR.7111.5p | 0.174764174 | 0.022465505 | 1.21E-14 |
| miR.1290 | 0.139065728 | 0.017960565 | 1.60E-14 |
| miR.320c | -0.195490025 | 0.025327354 | 1.92E-14 |
| miR.4690.5p | 0.206073535 | 0.027372227 | 8.00E-14 |
| miR.320a | -0.164056481 | 0.02246837 | 4.22E-13 |
| miR.191.5p | -0.188610635 | 0.025876716 | 4.63E-13 |
| miR.22.3p | -0.116127956 | 0.016017018 | 6.10E-13 |
| miR.149.3p | 0.198388481 | 0.027438381 | 7.04E-13 |
| miR.320b | -0.174898166 | 0.024221871 | 7.55E-13 |
| miR.6124 | 0.156186698 | 0.0223201 | 3.64E-12 |
| miR.6845.5p | 0.172094815 | 0.024612425 | 3.78E-12 |
| miR.4292 | 0.226056742 | 0.034321719 | 5.87E-11 |
| miR.134.5p | -0.082924778 | 0.012851764 | 1.40E-10 |
| miR.4534 | 0.216888225 | 0.034561225 | 4.34E-10 |
| miR.2861 | 0.130395813 | 0.020783001 | 4.37E-10 |
| miR.658 | -0.169393585 | 0.02704256 | 4.66E-10 |
| miR.6803.5p | 0.155141664 | 0.025048043 | 7.23E-10 |
| miR.5196.5p | 0.184406123 | 0.029813331 | 7.62E-10 |
| miR.7150 | 0.176862051 | 0.029946138 | 4.17E-09 |
| miR.24.3p | -0.176049721 | 0.02992356 | 4.77E-09 |
| miR.382.5p | -0.063871286 | 0.010903054 | 5.54E-09 |
| miR.130a.3p | -0.112420045 | 0.019315635 | 6.92E-09 |
| miR.409.3p | -0.063500942 | 0.011010897 | 9.44E-09 |
| miR.6131 | -0.105294718 | 0.018302048 | 1.02E-08 |
| miR.4644 | 0.077194729 | 0.01367532 | 1.91E-08 |
| miR.7107.5p | 0.119799725 | 0.021225893 | 1.92E-08 |
| miR.185.3p | -0.081409266 | 0.014571493 | 2.66E-08 |
| miR.1287.5p | -0.090970496 | 0.016406643 | 3.37E-08 |
| let.7g.5p | 0.218401587 | 0.039788037 | 4.60E-08 |
| miR.187.3p | -0.274722277 | 0.050280793 | 5.30E-08 |
| miR.151a.3p | -0.14393983 | 0.026932782 | 1.02E-07 |
| miR.4433b.5p | -0.167161368 | 0.031277677 | 1.02E-07 |
| miR.33b.5p | -0.210186166 | 0.039612116 | 1.26E-07 |
| miR.6852.5p | -0.161908967 | 0.030711216 | 1.51E-07 |
| miR.148a.3p | -0.129231877 | 0.024522394 | 1.53E-07 |
| miR.6088 | 0.133584257 | 0.025573857 | 1.96E-07 |
| miR.6821.5p | 0.111275785 | 0.021757142 | 3.48E-07 |
| miR.338.3p | -0.13985387 | 0.027438014 | 3.80E-07 |
| miR.425.3p | -0.095796042 | 0.018793443 | 3.80E-07 |
| miR.339.5p | -0.13809558 | 0.027224542 | 4.32E-07 |
| miR.320e | -0.128542339 | 0.025521686 | 5.20E-07 |
| miR.6778.5p | 0.177725253 | 0.035311078 | 5.30E-07 |
| miR.199a.3p | -0.119151376 | 0.023755544 | 5.79E-07 |
| miR.6870.5p | 0.10034671 | 0.02001533 | 5.86E-07 |
| miR.4632.5p | 0.086712536 | 0.017348026 | 6.33E-07 |
| miR.143.3p | -0.13889696 | 0.027880724 | 6.89E-07 |
| miR.103a.3p | -0.143742866 | 0.029298574 | 1.01E-06 |
| miR.370.3p | -0.07787291 | 0.016049375 | 1.33E-06 |
| miR.23b.3p | -0.147787831 | 0.030554433 | 1.43E-06 |
| miR.93.5p | -0.128978202 | 0.0267264 | 1.51E-06 |
| miR.4655.3p | -0.218275856 | 0.04583242 | 2.06E-06 |
| miR.3912.3p | -0.187330022 | 0.039870805 | 2.82E-06 |
| miR.584.5p | -0.052925606 | 0.011395488 | 3.65E-06 |
| miR.328.3p | -0.112597262 | 0.024261209 | 3.71E-06 |
| miR.199a.5p | -0.092511711 | 0.019993384 | 3.97E-06 |
| miR.4478 | 0.126441025 | 0.0273608 | 4.08E-06 |
| miR.484 | -0.137947355 | 0.029913351 | 4.27E-06 |
| miR.7845.5p | 0.102376753 | 0.022486105 | 5.64E-06 |
| miR.652.3p | -0.119910463 | 0.026439199 | 6.12E-06 |
| miR.1275 | 0.117007581 | 0.026191336 | 8.40E-06 |
| miR.31.5p | 0.094355321 | 0.021189445 | 8.98E-06 |
| miR.7106.5p | 0.084986054 | 0.019116271 | 9.28E-06 |
| miR.185.5p | -0.074353503 | 0.016936496 | 1.20E-05 |
| miR.107 | -0.13873327 | 0.031910153 | 1.45E-05 |
| miR.6794.5p | 0.252026951 | 0.058074841 | 1.50E-05 |
| miR.8069 | 0.117176767 | 0.027020976 | 1.53E-05 |
| miR.7108.5p | 0.116189592 | 0.026888122 | 1.63E-05 |
| miR.4447 | -0.074916257 | 0.017597836 | 2.18E-05 |
| miR.6794.3p | -0.209925139 | 0.04983101 | 2.65E-05 |

Table 1.2: Significant miRNAs for PLR after Bonferroni corrected threshold of P < 2.82x10^−5^.

| **miRNA** | **Beta** | **Se** | **P** |
| --- | --- | --- | --- |
| miR.654.5p | 0.32538233 | 0.017535675 | 1.42E-70 |
| miR.150.5p | 0.332590125 | 0.019257358 | 4.82E-62 |
| miR.6124 | 0.259389603 | 0.016995303 | 1.33E-49 |
| miR.1915.3p | 0.24470777 | 0.016625285 | 1.95E-46 |
| miR.29a.3p | 0.407792674 | 0.027869652 | 6.12E-46 |
| miR.3940.5p | 0.204376909 | 0.014370981 | 1.28E-43 |
| miR.5196.5p | 0.317354729 | 0.022859736 | 9.65E-42 |
| miR.7111.5p | 0.238935918 | 0.01734001 | 3.54E-41 |
| miR.6780b.5p | 0.217602171 | 0.015969532 | 2.41E-40 |
| miR.4449 | 0.21182969 | 0.015993856 | 2.63E-38 |
| miR.4534 | 0.333642808 | 0.026755005 | 2.56E-34 |
| miR.6085 | 0.178190603 | 0.014611545 | 5.99E-33 |
| miR.6845.5p | 0.226713363 | 0.019177685 | 3.94E-31 |
| miR.6798.5p | 0.140355253 | 0.012152298 | 7.76E-30 |
| miR.3648 | 0.238937515 | 0.020758701 | 1.19E-29 |
| miR.7150 | 0.267047025 | 0.023295992 | 1.98E-29 |
| miR.6727.5p | 0.163950155 | 0.014561353 | 1.75E-28 |
| miR.6803.5p | 0.219801924 | 0.019527137 | 1.81E-28 |
| miR.342.3p | 0.285414036 | 0.02564404 | 6.87E-28 |
| miR.4433b.5p | -0.260023837 | 0.024401774 | 9.12E-26 |
| miR.6778.5p | 0.286385949 | 0.027564267 | 1.31E-24 |
| miR.6799.5p | 0.210549373 | 0.020412969 | 2.75E-24 |
| miR.6870.5p | 0.160385983 | 0.015634292 | 4.77E-24 |
| miR.4292 | 0.275916834 | 0.026943013 | 5.68E-24 |
| miR.149.3p | 0.221042537 | 0.021590814 | 5.84E-24 |
| miR.1237.5p | 0.218466559 | 0.02156368 | 1.65E-23 |
| miR.7107.5p | 0.167895029 | 0.016623687 | 2.23E-23 |
| miR.223.3p | -0.167136662 | 0.01704532 | 3.70E-22 |
| miR.4478 | 0.205720136 | 0.021422253 | 2.44E-21 |
| miR.326 | -0.141845482 | 0.014881561 | 4.71E-21 |
| miR.155.5p | 0.167029017 | 0.017817332 | 1.97E-20 |
| miR.6088 | 0.184721707 | 0.02009545 | 1.01E-19 |
| miR.4690.5p | 0.197690045 | 0.021685617 | 1.98E-19 |
| miR.6821.5p | 0.152011318 | 0.017116272 | 1.54E-18 |
| miR.4655.3p | -0.319067147 | 0.036026628 | 1.91E-18 |
| miR.6894.5p | 0.160559908 | 0.018154413 | 2.12E-18 |
| miR.187.3p | -0.348727869 | 0.039609079 | 2.98E-18 |
| miR.4429 | -0.172390745 | 0.019591021 | 3.11E-18 |
| miR.29c.3p | 0.260819434 | 0.029799623 | 4.64E-18 |
| miR.4430 | 0.124087917 | 0.014449625 | 1.86E-17 |
| miR.320d | -0.16250367 | 0.01904098 | 2.91E-17 |
| miR.22.3p | -0.107882187 | 0.012712469 | 4.33E-17 |
| miR.320c | -0.170402479 | 0.020141589 | 5.37E-17 |
| miR.8069 | 0.178173679 | 0.021263452 | 1.04E-16 |
| miR.6086 | 0.087854201 | 0.010572641 | 1.84E-16 |
| miR.320a | -0.147728681 | 0.017852962 | 2.45E-16 |
| miR.29b.3p | 0.227578839 | 0.027718315 | 4.11E-16 |
| miR.1275 | 0.167954113 | 0.020638624 | 7.34E-16 |
| miR.320b | -0.156642589 | 0.019251743 | 7.42E-16 |
| miR.33b.5p | -0.253750052 | 0.031286714 | 9.13E-16 |
| miR.2861 | 0.132674928 | 0.016468091 | 1.40E-15 |
| miR.1290 | 0.11526497 | 0.014309714 | 1.42E-15 |
| miR.4270 | 0.163122359 | 0.020670988 | 5.10E-15 |
| miR.15a.5p | -0.203900154 | 0.026036683 | 8.11E-15 |
| miR.7108.5p | 0.165547505 | 0.021209966 | 9.91E-15 |
| miR.3912.3p | -0.243874371 | 0.031487633 | 1.57E-14 |
| miR.6869.5p | 0.17384327 | 0.023009044 | 6.55E-14 |
| miR.4644 | 0.08151897 | 0.010838183 | 8.43E-14 |
| miR.6781.5p | 0.12809485 | 0.017424914 | 2.94E-13 |
| miR.15b.5p | -0.166348908 | 0.022972344 | 6.51E-13 |
| miR.130a.3p | -0.110506835 | 0.015335436 | 8.38E-13 |
| miR.658 | -0.154195343 | 0.021503843 | 1.08E-12 |
| miR.93.5p | -0.150175454 | 0.02116787 | 1.85E-12 |
| miR.2115.5p | -0.275510423 | 0.038871782 | 1.94E-12 |
| miR.6775.5p | 0.139674452 | 0.019798825 | 2.44E-12 |
| miR.6870.3p | -0.125189027 | 0.018008335 | 5.00E-12 |
| miR.4640.3p | 0.105881018 | 0.01524231 | 5.18E-12 |
| miR.24.3p | -0.164491922 | 0.023788119 | 6.44E-12 |
| miR.7106.5p | 0.104476074 | 0.015136938 | 7.04E-12 |
| miR.4279 | -0.223688209 | 0.032505937 | 8.10E-12 |
| miR.497.3p | -0.208160964 | 0.030300848 | 8.77E-12 |
| miR.4459 | 0.169753893 | 0.024788672 | 1.02E-11 |
| miR.6794.3p | -0.269435718 | 0.039446799 | 1.15E-11 |
| miR.6742.5p | -0.21377393 | 0.03151148 | 1.57E-11 |
| miR.134.5p | -0.069460017 | 0.010240776 | 1.59E-11 |
| miR.212.3p | -0.239307807 | 0.03622899 | 5.18E-11 |
| miR.185.5p | -0.088186474 | 0.013425165 | 6.59E-11 |
| miR.5703 | 0.110073519 | 0.016861285 | 8.59E-11 |
| miR.765 | 0.121970922 | 0.018927594 | 1.48E-10 |
| miR.2115.3p | -0.236221871 | 0.036813981 | 1.77E-10 |
| miR.103a.3p | -0.148242972 | 0.023270238 | 2.38E-10 |
| miR.382.5p | -0.055059072 | 0.008684438 | 2.89E-10 |
| miR.3912.5p | -0.219729511 | 0.03487194 | 3.69E-10 |
| miR.3140.5p | -0.240875597 | 0.038292176 | 3.95E-10 |
| miR.937.3p | -0.198908432 | 0.031648782 | 4.09E-10 |
| miR.6797.5p | 0.083653755 | 0.013446275 | 6.09E-10 |
| miR.6812.5p | 0.136368228 | 0.02194587 | 6.38E-10 |
| miR.191.5p | -0.127753049 | 0.020726861 | 8.72E-10 |
| miR.23b.3p | -0.149650104 | 0.024280099 | 8.73E-10 |
| miR.504.3p | 0.087653785 | 0.014312375 | 1.11E-09 |
| miR.451a | -0.068175363 | 0.011161771 | 1.23E-09 |
| miR.7845.5p | 0.108338801 | 0.017861484 | 1.59E-09 |
| miR.4728.5p | 0.087492207 | 0.014433116 | 1.63E-09 |
| miR.640 | -0.203063503 | 0.033538823 | 1.70E-09 |
| miR.4309 | -0.198606611 | 0.033365939 | 3.16E-09 |
| miR.4632.5p | 0.081879662 | 0.013801817 | 3.56E-09 |
| miR.181c.5p | -0.185132632 | 0.031310765 | 4.00E-09 |
| miR.648 | -0.200693773 | 0.034037812 | 4.42E-09 |
| miR.6771.5p | 0.076409867 | 0.01311322 | 6.65E-09 |
| miR.6852.5p | -0.142271718 | 0.024460753 | 7.08E-09 |
| miR.3652 | 0.069668661 | 0.012032466 | 8.26E-09 |
| miR.3180 | -0.088886422 | 0.015376763 | 8.73E-09 |
| miR.199a.3p | -0.108199813 | 0.018912896 | 1.23E-08 |
| miR.16.5p | -0.125566926 | 0.022030922 | 1.40E-08 |
| miR.107 | -0.143428048 | 0.025367559 | 1.81E-08 |
| miR.143.5p | -0.157093463 | 0.027830053 | 1.91E-08 |
| miR.187.5p | -0.196242936 | 0.034874672 | 2.12E-08 |
| miR.5739 | -0.06401119 | 0.011475721 | 2.80E-08 |
| miR.4707.3p | 0.139624737 | 0.02503911 | 2.82E-08 |
| miR.374b.3p | -0.162756134 | 0.029366187 | 3.42E-08 |
| miR.6741.5p | 0.092483171 | 0.016700999 | 3.51E-08 |
| miR.151a.3p | -0.11850944 | 0.021475168 | 3.91E-08 |
| miR.3180.3p | -0.091857765 | 0.016674554 | 4.12E-08 |
| miR.484 | -0.130709033 | 0.02380697 | 4.57E-08 |
| miR.339.5p | -0.119033345 | 0.021693299 | 4.65E-08 |
| miR.1307.5p | -0.100572006 | 0.01834729 | 4.80E-08 |
| miR.6809.5p | -0.16054753 | 0.02932141 | 4.97E-08 |
| miR.34b.3p | -0.144805833 | 0.026540797 | 5.53E-08 |
| miR.5694 | -0.144096519 | 0.026424095 | 5.62E-08 |
| miR.6794.5p | 0.251279321 | 0.046196277 | 6.06E-08 |
| miR.210.3p | -0.200919572 | 0.037032881 | 6.55E-08 |
| miR.6880.5p | 0.12559249 | 0.023196442 | 6.96E-08 |
| miR.1307.3p | -0.09400497 | 0.017382034 | 7.20E-08 |
| miR.3184.3p | -0.083550472 | 0.0154586 | 7.34E-08 |
| miR.561.5p | -0.134641266 | 0.024936372 | 7.56E-08 |
| miR.1287.5p | -0.070684585 | 0.0130943 | 7.61E-08 |
| miR.34a.5p | 0.088624236 | 0.016501364 | 8.84E-08 |
| miR.4291 | -0.139973737 | 0.026237993 | 1.08E-07 |
| miR.3197 | 0.040409953 | 0.007691301 | 1.66E-07 |
| miR.652.3p | -0.110176785 | 0.021053644 | 1.86E-07 |
| miR.199a.5p | -0.083199057 | 0.015924811 | 1.95E-07 |
| miR.1247.3p | -0.096834512 | 0.018584219 | 2.09E-07 |
| miR.409.3p | -0.045707764 | 0.008798918 | 2.28E-07 |
| miR.29c.5p | 0.079506851 | 0.015439026 | 2.89E-07 |
| miR.3162.5p | 0.06274266 | 0.012223545 | 3.15E-07 |
| miR.4656 | 0.064371424 | 0.012657742 | 4.04E-07 |
| miR.17.5p | -0.140054858 | 0.027686614 | 4.65E-07 |
| miR.376c.3p | -0.084669381 | 0.016794524 | 5.07E-07 |
| miR.217 | -0.129017777 | 0.025620357 | 5.23E-07 |
| miR.381.3p | -0.088656205 | 0.017711169 | 6.10E-07 |
| miR.7851.3p | 0.085233083 | 0.017068872 | 6.49E-07 |
| miR.320e | -0.100571885 | 0.02036571 | 8.60E-07 |
| miR.6804.3p | -0.183826899 | 0.037255848 | 8.78E-07 |
| miR.4505 | 0.188878606 | 0.038320889 | 9.02E-07 |
| miR.23a.3p | -0.107670456 | 0.021873586 | 9.31E-07 |
| miR.6131 | -0.071845131 | 0.014636085 | 9.98E-07 |
| miR.764 | -0.097852021 | 0.020064542 | 1.17E-06 |
| miR.1249 | -0.154248548 | 0.03165783 | 1.20E-06 |
| miR.425.3p | -0.07247466 | 0.01500346 | 1.47E-06 |
| miR.361.3p | 0.047417697 | 0.009823607 | 1.50E-06 |
| miR.197.5p | -0.05335347 | 0.011060663 | 1.53E-06 |
| miR.6771.3p | -0.218766392 | 0.045733816 | 1.86E-06 |
| miR.4306 | -0.08920305 | 0.018706453 | 2.00E-06 |
| miR.6785.3p | -0.189218598 | 0.040169153 | 2.66E-06 |
| miR.126.3p | -0.108969514 | 0.023192794 | 2.82E-06 |
| miR.2116.5p | -0.144249766 | 0.03077204 | 2.97E-06 |
| miR.6738.5p | 0.082248711 | 0.017607119 | 3.21E-06 |
| miR.148b.3p | -0.083113467 | 0.017874554 | 3.56E-06 |
| miR.3157.5p | -0.139246447 | 0.029991226 | 3.68E-06 |
| miR.6789.5p | -0.093418206 | 0.020287565 | 4.41E-06 |
| miR.30d.5p | -0.163704377 | 0.03558654 | 4.51E-06 |
| miR.4758.5p | 0.104335506 | 0.02269561 | 4.58E-06 |
| miR.670.3p | -0.140377913 | 0.030720539 | 5.21E-06 |
| miR.136.5p | -0.032500518 | 0.00713199 | 5.53E-06 |
| miR.148a.3p | -0.089120856 | 0.01960054 | 5.80E-06 |
| miR.4496 | 0.052157454 | 0.011474808 | 5.84E-06 |
| miR.6782.5p | 0.050748366 | 0.01118793 | 6.10E-06 |
| miR.19a.3p | -0.094055418 | 0.020745905 | 6.17E-06 |
| miR.4695.5p | 0.08462687 | 0.018856049 | 7.63E-06 |
| miR.3135a | 0.092980227 | 0.020759901 | 7.97E-06 |
| miR.3140.3p | -0.154534593 | 0.03458586 | 8.37E-06 |
| miR.185.3p | -0.052023562 | 0.011660039 | 8.62E-06 |
| miR.186.5p | -0.131863054 | 0.029876411 | 1.08E-05 |
| miR.4800.5p | 0.094943911 | 0.021627085 | 1.20E-05 |
| miR.935 | -0.068657228 | 0.015777818 | 1.43E-05 |
| miR.370.3p | -0.055528198 | 0.012820164 | 1.56E-05 |
| miR.4795.5p | -0.113673139 | 0.026246535 | 1.56E-05 |
| miR.6875.5p | 0.044430604 | 0.010322744 | 1.76E-05 |
| miR.376a.3p | -0.047787068 | 0.011168342 | 1.98E-05 |
| miR.561.3p | -0.077540121 | 0.018149997 | 2.03E-05 |
| miR.3943 | -0.091265557 | 0.021394503 | 2.09E-05 |
| miR.221.3p | -0.066408236 | 0.015617087 | 2.22E-05 |
| miR.6729.3p | -0.1628193 | 0.038290883 | 2.22E-05 |
| miR.6796.3p | -0.0874708 | 0.020604777 | 2.29E-05 |
| miR.130b.3p | -0.063100967 | 0.014885995 | 2.36E-05 |
| miR.1233.3p | -0.074388072 | 0.017618784 | 2.54E-05 |
| miR.374b.5p | -0.100580971 | 0.023875898 | 2.65E-05 |

Table 1.3: Significant miRNAs for NLR after Bonferroni corrected threshold of P < 2.82x10^−5^.

| **miRNA** | **Beta** | **se** | **P** |
| --- | --- | --- | --- |
| miR.150.5p | 0.367447027 | 0.019231531 | 2.32E-74 |
| miR.29a.3p | 0.425619774 | 0.028167053 | 1.04E-48 |
| miR.654.5p | 0.256602695 | 0.01843242 | 5.92E-42 |
| miR.342.3p | 0.31647246 | 0.025835421 | 3.23E-33 |
| miR.223.3p | -0.205297522 | 0.017074081 | 4.14E-32 |
| miR.29c.3p | 0.322005639 | 0.029916344 | 3.05E-26 |
| miR.3940.5p | 0.157966687 | 0.014907768 | 1.67E-25 |
| miR.4449 | 0.163656602 | 0.016542935 | 1.62E-22 |
| miR.6780b.5p | 0.160935136 | 0.01657531 | 9.10E-22 |
| miR.29b.3p | 0.270490074 | 0.027917645 | 1.10E-21 |
| miR.155.5p | 0.174674816 | 0.01804249 | 1.18E-21 |
| miR.1915.3p | 0.163537677 | 0.017415301 | 1.71E-20 |
| miR.6727.5p | 0.1357487 | 0.014937004 | 2.54E-19 |
| miR.149.3p | 0.195732665 | 0.022045087 | 1.57E-18 |
| miR.6085 | 0.132178552 | 0.015095502 | 4.50E-18 |
| miR.1237.5p | 0.192152207 | 0.022020531 | 5.81E-18 |
| miR.6798.5p | 0.102018901 | 0.012540656 | 7.51E-16 |
| miR.187.3p | -0.323492615 | 0.040309029 | 1.79E-15 |
| miR.4690.5p | 0.170536153 | 0.02213038 | 2.11E-14 |
| let.7g.5p | 0.245049505 | 0.031946466 | 2.76E-14 |
| miR.658 | -0.166737279 | 0.021766809 | 2.99E-14 |
| miR.7150 | 0.184232102 | 0.024076687 | 3.18E-14 |
| miR.3648 | 0.160445904 | 0.021475786 | 1.22E-13 |
| miR.4655.3p | -0.27120572 | 0.036769094 | 2.46E-13 |
| miR.4433b.5p | -0.178390696 | 0.025159792 | 1.90E-12 |
| miR.6803.5p | 0.142131249 | 0.020205858 | 2.82E-12 |
| miR.6845.5p | 0.139303459 | 0.019913054 | 3.69E-12 |
| miR.4534 | 0.191520655 | 0.027905175 | 9.18E-12 |
| miR.6821.5p | 0.118658061 | 0.017510396 | 1.65E-11 |
| miR.2861 | 0.113622066 | 0.016786133 | 1.74E-11 |
| miR.33b.5p | -0.21544122 | 0.031900009 | 1.93E-11 |
| miR.6124 | 0.121700312 | 0.018076109 | 2.22E-11 |
| miR.1225.3p | -0.205580093 | 0.031070317 | 4.81E-11 |
| miR.4292 | 0.182327189 | 0.027770898 | 6.74E-11 |
| miR.6088 | 0.13476627 | 0.020605609 | 7.94E-11 |
| miR.1290 | 0.09501794 | 0.014599415 | 9.78E-11 |
| miR.1287.5p | -0.086042596 | 0.013233357 | 1.02E-10 |
| miR.7111.5p | 0.11836666 | 0.018265798 | 1.17E-10 |
| miR.3912.3p | -0.205359299 | 0.0320958 | 1.99E-10 |
| miR.5196.5p | 0.152758084 | 0.024109078 | 2.96E-10 |
| miR.7107.5p | 0.106329868 | 0.017142979 | 6.85E-10 |
| miR.4429 | -0.117994596 | 0.02009583 | 5.11E-09 |
| miR.4478 | 0.129322082 | 0.022059731 | 5.40E-09 |
| miR.4644 | 0.063981207 | 0.011059612 | 8.50E-09 |
| miR.361.3p | 0.054663866 | 0.009944364 | 4.41E-08 |
| miR.3197 | 0.04266323 | 0.007795418 | 5.04E-08 |
| miR.361.5p | 0.135527943 | 0.024784467 | 5.17E-08 |
| miR.6804.3p | -0.203955829 | 0.037734592 | 7.33E-08 |
| miR.574.3p | -0.128003911 | 0.023700346 | 7.49E-08 |
| miR.1255b.2.3p | -0.08628813 | 0.016022908 | 8.16E-08 |
| miR.320c | -0.111116326 | 0.02065961 | 8.48E-08 |
| miR.4632.5p | 0.075127915 | 0.014021824 | 9.48E-08 |
| miR.320d | -0.104352302 | 0.019539279 | 1.04E-07 |
| miR.1207.5p | -0.12798679 | 0.024081979 | 1.20E-07 |
| miR.195.5p | 0.132731471 | 0.025186302 | 1.53E-07 |
| miR.1202 | -0.237013846 | 0.045000778 | 1.55E-07 |
| miR.6742.5p | -0.168444601 | 0.032116724 | 1.75E-07 |
| miR.6869.5p | 0.123164388 | 0.023520419 | 1.82E-07 |
| miR.1285.5p | -0.076263564 | 0.01473092 | 2.50E-07 |
| miR.3180 | -0.080084248 | 0.015624659 | 3.28E-07 |
| miR.6894.5p | 0.094761951 | 0.018669619 | 4.25E-07 |
| miR.187.5p | -0.178289315 | 0.035429308 | 5.32E-07 |
| miR.5703 | 0.086288515 | 0.017180099 | 5.59E-07 |
| miR.2115.5p | -0.198651243 | 0.03968894 | 6.11E-07 |
| miR.6870.5p | 0.081060169 | 0.016194258 | 6.11E-07 |
| miR.6778.5p | 0.142243316 | 0.028573551 | 7.02E-07 |
| miR.5739 | -0.058027349 | 0.01165823 | 7.05E-07 |
| miR.6729.3p | -0.191712513 | 0.038767034 | 8.30E-07 |
| miR.1247.3p | -0.09251419 | 0.018863366 | 1.02E-06 |
| miR.3180.3p | -0.083041585 | 0.016939649 | 1.03E-06 |
| miR.3912.5p | -0.174120854 | 0.035515067 | 1.03E-06 |
| miR.326 | -0.075279493 | 0.015361578 | 1.04E-06 |
| miR.6892.3p | -0.117592601 | 0.024048 | 1.10E-06 |
| miR.31.5p | 0.083698041 | 0.017125122 | 1.11E-06 |
| miR.4291 | -0.129434018 | 0.026644741 | 1.29E-06 |
| miR.7106.5p | 0.07493303 | 0.01545086 | 1.34E-06 |
| miR.5694 | -0.129567477 | 0.026845122 | 1.50E-06 |
| miR.320b | -0.094891899 | 0.01975002 | 1.68E-06 |
| miR.574.5p | -0.081374911 | 0.016971958 | 1.76E-06 |
| miR.142.5p | 0.093444521 | 0.01952254 | 1.83E-06 |
| miR.4270 | 0.101355656 | 0.021185174 | 1.85E-06 |
| miR.34b.3p | -0.128961706 | 0.026966988 | 1.87E-06 |
| miR.6781.5p | 0.084862208 | 0.017820564 | 2.07E-06 |
| miR.320a | -0.087018962 | 0.018328646 | 2.22E-06 |
| miR.4279 | -0.157201612 | 0.033187241 | 2.34E-06 |
| miR.1273e | -0.07065501 | 0.014920926 | 2.36E-06 |
| miR.6809.5p | -0.140390123 | 0.029799364 | 2.65E-06 |
| miR.6775.3p | -0.201439313 | 0.042776244 | 2.67E-06 |
| miR.29c.5p | 0.073353079 | 0.015677397 | 3.10E-06 |
| miR.6794.3p | -0.18776578 | 0.040273681 | 3.35E-06 |
| miR.877.3p | -0.130426492 | 0.02803416 | 3.52E-06 |
| miR.3184.3p | -0.072842223 | 0.015710294 | 3.79E-06 |
| miR.22.3p | -0.060452244 | 0.013067355 | 3.98E-06 |
| miR.1307.5p | -0.085817669 | 0.018651851 | 4.49E-06 |
| miR.7114.3p | -0.111062612 | 0.024592355 | 6.70E-06 |
| miR.1304.3p | -0.119502362 | 0.026470613 | 6.75E-06 |
| miR.1233.3p | -0.079056273 | 0.017860026 | 1.01E-05 |
| miR.6785.3p | -0.179472002 | 0.040769465 | 1.13E-05 |
| miR.210.3p | -0.165329044 | 0.037660701 | 1.20E-05 |
| miR.497.3p | -0.135496748 | 0.030962032 | 1.28E-05 |
| miR.197.5p | -0.048952176 | 0.01123037 | 1.38E-05 |
| miR.3652 | 0.053397269 | 0.012250776 | 1.38E-05 |
| miR.7108.5p | 0.094708557 | 0.021752854 | 1.41E-05 |
| miR.1275 | 0.092197309 | 0.021196702 | 1.44E-05 |
| miR.6131 | -0.064375393 | 0.014864946 | 1.57E-05 |
| miR.640 | -0.147689915 | 0.034178978 | 1.64E-05 |
| miR.7845.5p | 0.078001167 | 0.018204588 | 1.92E-05 |
| miR.4447 | -0.060796008 | 0.014237548 | 2.05E-05 |
| miR.212.3p | -0.156996335 | 0.03699587 | 2.31E-05 |
| miR.6796.3p | -0.088264181 | 0.020897899 | 2.52E-05 |
| miR.181c.5p | -0.134590303 | 0.031901424 | 2.57E-05 |
| miR.2115.3p | -0.158538556 | 0.037570675 | 2.57E-05 |
| miR.6771.3p | -0.194979026 | 0.046447969 | 2.82E-05 |


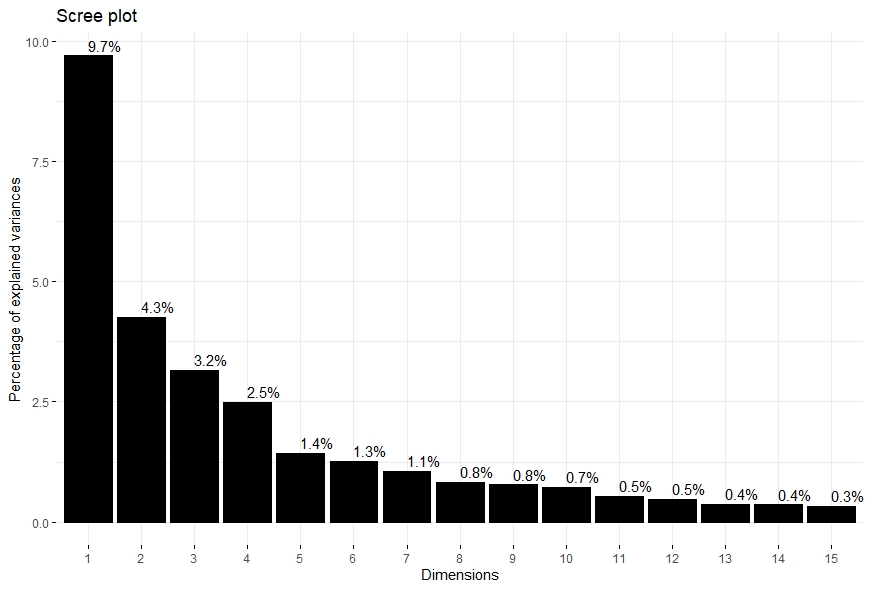


**Figure S1: Scree plot depicting the first 15 principal components and their respective explained variances.** The first two principal components are used for the principal component regression analysis.


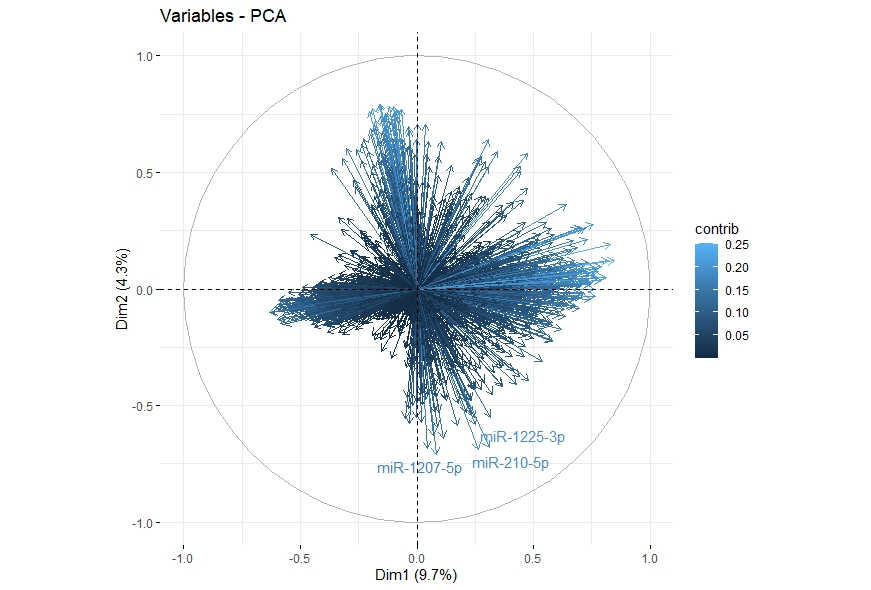


**Figure S2: Variable contribution of PC1 and PC2.** Dim1 is the percentage of explained variance for PC1 and Dim2 is the percentage of explained variance for PC2.

Table S3.1: Genes significantly associated with SII after the Bonferroni-corrected threshold of P < 1.52x10^-3^.

| **Gene** | **Probe ID** | **Beta** | **se** | **P** |
| --- | --- | --- | --- | --- |
| IKZF3 | ILMN_2300695 | -0.12522 | 0.013649 | 3.19E-19 |
| IKZF3 | ILMN_1669692 | -0.10038 | 0.014289 | 4.31E-12 |
| RPH3A | ILMN_1663356 | 0.086439 | 0.024536 | 4.49E-04 |
| EPS15L1 | ILMN_1708369 | 0.096937 | 0.02084 | 3.80E-06 |
| PTPN4 | ILMN_1793549 | -0.14572 | 0.012906 | 1.08E-27 |
| ATP6V1G3 | ILMN_1726273 | 0.086608 | 0.026896 | 1.33E-03 |
| STRN | ILMN_1696190 | 0.109973 | 0.015746 | 5.67E-12 |

Table S3.2: Genes significantly associated with PLR at the Bonferroni-corrected threshold of P < 1.52x10^-3^.

| **Gene** | **Probe ID** | **Beta** | **se** | **P** |
| --- | --- | --- | --- | --- |
| IKZF3 | ILMN_2300695 | -0.07422 | 0.009799 | 9.18E-14 |
| IKZF3 | ILMN_1669692 | -0.04573 | 0.01028 | 9.78E-06 |
| RPH3A | ILMN_1663356 | 0.060636 | 0.017368 | 5.05E-04 |
| EPS15L1 | ILMN_1708369 | 0.05102 | 0.014832 | 6.09E-04 |
| PTPN4 | ILMN_1793549 | -0.10735 | 0.00908 | 5.00E-30 |
| STRN | ILMN_1696190 | 0.08319 | 0.011101 | 1.63E-13 |
| STRN | ILMN_1749882 | 0.067717 | 0.011336 | 3.38E-09 |

Table S3.3: Genes significantly associated with NLR at the Bonferroni-corrected threshold of P < 1.52x10^-3^.

| **Gene** | **Probe ID** | **Beta** | **se** | **P** |
| --- | --- | --- | --- | --- |
| IKZF3 | ILMN_2300695 | -0.11872 | 0.011448 | 7.56E-24 |
| IKZF3 | ILMN_1669692 | -0.07975 | 0.012175 | 9.77E-11 |
| RPH3A | ILMN_1663356 | 0.078799 | 0.020775 | 1.59E-04 |
| EPS15L1 | ILMN_1708369 | 0.086188 | 0.017656 | 1.25E-06 |
| PTPN4 | ILMN_1793549 | -0.12694 | 0.010900 | 3.02E-29 |
| ATP6V1G3 | ILMN_1726273 | 0.075570 | 0.022831 | 9.71E-04 |
| STRN | ILMN_1696190 | 0.093171 | 0.013373 | 6.35E-12 |
| STRN | ILMN_1749882 | 0.051777 | 0.013762 | 1.79E-04 |
